# Sex-Related Gene Network Revealed by Transcriptome Differentiation of Bisexual and Unisexual Flowers of Orchid *Cymbidium tortisepalum*

**DOI:** 10.3390/ijms242316627

**Published:** 2023-11-22

**Authors:** Xiaokai Ma, Song Ju, Han Lin, Huaxing Huang, Jie Huang, Donghui Peng, Ray Ming, Siren Lan, Zhong-Jian Liu

**Affiliations:** 1 Center for Genomics and Biotechnology, Haixia Institute of Science and Technology, School of Future Technology, Fujian Agriculture and Forestry University, Fuzhou 350002, China; 2 Key Laboratory of Orchid Conservation and Utilization of National Forestry and Grassland Administration at College of Landscape Architecture, Fujian Agriculture and Forestry University, Fuzhou 350002, China; 3 Department of Plant Biology, University of Illinois at Urbana-Champaign, Urbana, IL 61801-3707, USA

**Keywords:** sex separation, androecium, gynostemium, reproductive organs, female and male sterility, mutation

## Abstract

Despite extensive research on orchid reproductive strategies, the genetic studies of sex differentiation in the orchid family are still lacking. In this study, we compared three sexual phenotypes of *Cymbidium tortisepalum* bisexual flowers as well as female and male unisexual mutants. Through comparative transcriptomes, we analyzed the sex-biased differentially expressed genes (DEGs) and gene co-expression networks of sex organs (gynostemium and ovary) among them, identified the candidate genes of sex differentiation, and validated their expression by qRT-PCR. The *C. tortisepalum* unisexual mutants with degenerated phenotypes were compared to the bisexual plants with respect to both the flower organs and plant morphologies. Totally, 12,145, 10,789, and 14,447 genes were uniquely expressed in the female, male, and hermaphrodite sex organs, respectively. A total of 4291 sex-biased DEGs were detected among them, with 871, 2867, and 1937 DEGs in the comparisons of bisexual vs. female, bisexual vs. male, and male vs. female flowers, respectively. Two co-expressed network modules, with 81 and 419 genes were tightly correlated with female sexual traits, while two others with 265 and 135 genes were highly correlated with male sexual traits. Two female-biased hub genes (*CtSDR3b* and *CtSDR3b-like*) nested in the female modules, the homologs of maize sex determinant *tasselseed2*, may control the feminization of *C. tortisepalum*. At the same time, two male-biased hub genes (*CtYAB2* and *CtYAB5*) nested in the male modules, the homologs of grape sex determinant *VviYABBY3,* may control the androphany of *C. tortisepalum*. This study discovered the molecular regulation networks and proposed a model for orchid sex differentiation, therefore providing for the first time the genetic basis for the sex separation in the orchid family.

## 1. Introduction

Sex separation, as one type of mating systems, repeatedly and independently evolves during the process of reproduction diversification in land plants [1,2]. Sex differentiation controlled by factors such as sex determinants, sex chromosomes, plant hormones, and environmental stress is usually accompanied by the evolution of dioecy [3,4,5]. In dioecious plants, inbreeding depression can be avoided, and greater genetic variation as well as post-pollination fitness can be maintained by a high outcrossing rate and resource allocation [6,7,8]. Since the pathways of sex separation are not conserved in the plant kingdoms, while sex determination mechanisms diversify among different lineages [9], research on the mechanism of sex differentiation in plants is insufficient compared to that for animals [10]. Recently, with the progress of genetic theory and rapid development of genomic technologies, such as high-throughput sequencing, the mechanisms of plant sex differentiation include sex-determining factors [11,12], sex chromosomes [2,5,13,14] and hormone regulation [15], which has been reported extensively in several plant lineages during two most recent decades.

Unisexual characteristics often occur when one sex (either female or male) organ of a hermaphrodite is sterile and makes it evolve into a unisexual individual [5,16,17]. In the genus *Populus*, the loss of female function leads to the formation of male plants, and in individual male gametes, they are sterile without function, evolving into the differentiation of single female and male sexes [18,19,20]. The femaleness of the bisexual flowers of *Carica papaya* can inhibit male function to produce female flowers, while the male flowers evolve from female sterility [13]. The unisexual flowers of *Spinacia oleracea* occur at the initiation of primordia with the abortion of carpels in the male flowers or loss of stamens in the female flowers. Spinach male-specific gene *NRT1/PTR6.4* which might control stamen initiation/carpel suppression that evolved in the Y chromosome was proposed as a candidate for sex determination [5]. However, some lineages showed the opposite evolutionary pathways, evolving into hermaphrodites from a single independent sex. For example, the genus *Momordica* in Cucurbitaceae has undergone seven repeated and independent evolutionary transitions from dioecy to monoecy [21]. In addition, the expression of sex-determining genes in *Momordica* indirectly induces hormone differentiation in sexual organs, contributing to sex differentiation [21,22].

Orchidaceae, accounting for ~10% of flowering plants, is one of the largest families in angiosperms [23]. Orchids are renowned for their extraordinary and diversified floral morphologies, such as bilateral and inverted flowers, fused gynostemium (e.g., column, a fused organ of the androecium and gynoecium), and specialized labellum adaptation in morphology, color, and scent to specific pollinators [24,25]. The first whole genome of orchids was sequenced for *Phalaenopsis equestris*, which showed multiple MADS-box genes specifically amplified in orchids and associated with orchid flower morphologies [26]. By sequencing the genome of the orchid *Apostasia shenzhenica*, several expanded and contracted MADS-box genes were identified that might contribute to the key innovations of the orchid reproductive organs such as specialized pollinia, gynostemium, and labellum [27]. Although these studies provide the genomic basis of key floral innovations in the orchid family, few studies have explored the sexual system in orchids. Huang et al. [28] showed that the orchid *Satyrium ciliatum* has a gynodioecious sexual system with female population performing parthenogenesis that maintains male sterility. Another study of the orchid subtribe Catasetinae revealed that environmental sex determination (ESD) evolved three times for the sexual systems within it [29]. Despite several reports on the reproductive ecology as well the divergence of the sexual systems in orchids, the genetic basis for the sex differentiation/determination mechanisms in the orchid family remains unexplored.

**Figure 1:**
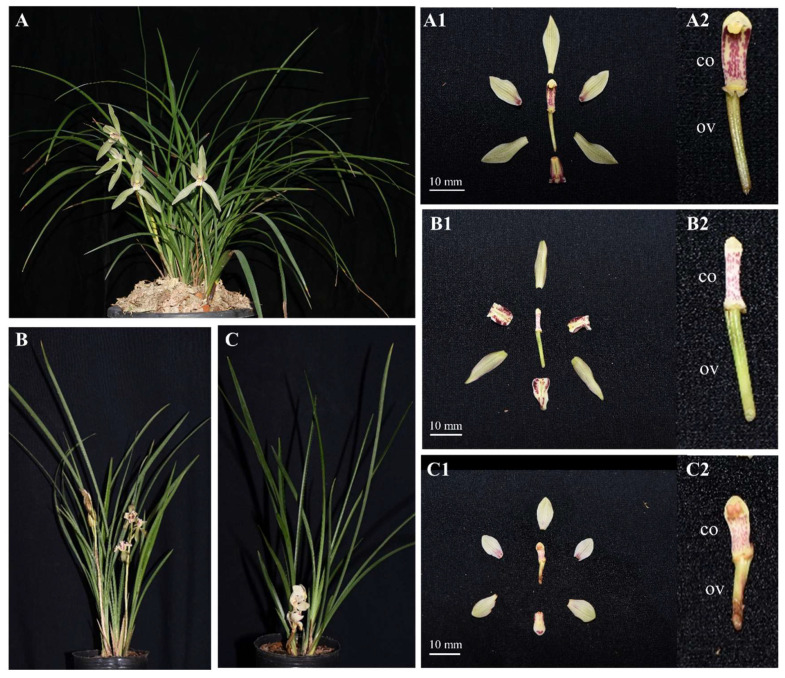
Sexual phenotypes (hermaphrodite, female, and male) of *C. tortisepalum* plants and flowers. (**A**) Hermaphroditic plant ((**A1**) hermaphroditic flower; (**A2**) hermaphroditic gynostemium column (CO) and ovary (OV)); (**B**) female plant ((**B1**) female flower; (**B2**) female gynostemium and ovary); (**C**) male plant ((**C1**) male flower; (**C2**) male gynostemium and ovary).

*Cymbidium tortisepalum* Fukuyama belongs to the genus *Cymbidium*, a terrestrial orchid [23]. In wild populations, *C. tortisepalum* rarely has male and female unisexual mutants, which provide excellent materials for investigating the mechanisms of sex differentiation or determination in orchids [30,31]. There are obvious morphological differences between normal hermaphrodites and female and male mutants in *C. tortisepalum*. The bisexual (hermaphrodite) flower has a normal gynostemium (e.g., column), a slender ovary, narrow elliptic petals, and a trifid labellum (Figure 1A). However, the female (male-sterile) flower has a feminized column with degenerated stamens without the anthers and pollinia but with a clear stigma cavity, narrower elliptical sepals, labellate petals, and an apiculate ovate labellum (Figure 1B). Comparatively, the male (female-sterile) flower has a stubby masculinized column with an underdeveloped stigma cavity, a short degenerated ovary, round obovate sepals and petals, and a small round labellum (Figure 1C).

To investigate the sex differentiation mechanisms in orchids, in this study, the bisexual, male, and female flowers of *C. tortisepalum* plants (Figure 1) were selected to construct the sex-related gene networks of the orchid flower *C. tortisepalum* using comparative transcriptomes, sex-specific expression profiles as well as co-expression networks. We firstly compared the floral structure, especially the morphology of the column (e.g., gynostemium) and ovary among three sexual phenotypes. Then, the transcriptomes of the mixed samples (column + ovary, abbreviated as COOV; Figure 1(A2–C2)) of the column and ovary were sequenced among them. The expression profiles and differentially expressed genes (DEGs) among the three sexual phenotypes were investigated. Lastly, the co-expression networks were constructed, the candidate genes underlying the sex differentiation were identified, and the expression patterns were validated by qRT–PCR. Our study provides the first genetic evidence of sex differentiation in orchids and further supports the theoretical basis for discovering novel sex determination pathways in angiosperms.

## 2. Results

### 2.1. Morphological Comparison of Sexual Phenotypes in C. tortisepalum

Compared to the hermaphrodite *C. tortisepalum* individuals, male mutants are robust with lower height and fewer short stout leaves, while female mutants are slim with higher height and fewer long slim leaves (Figure 1). The average plant height among the three sexual phenotypes was different, with female (725.00 ± 53.45 mm, mean ± s.d.) and hermaphrodite (697 ± 36.04 mm) plants taller than male (592 ± 21.93 mm) plants (*t*-test, both *p* < 0.05; Table S1; Figure 1A–C).

The bisexual flowers in the hermaphroditic plants showed narrow elliptic petals, slightly trifid labella (LB), long curled columns (CO) densely covered with purple lines and dots, obvious stigmas, and slender ovaries (OV) (Figure 1(A1,A2)). There is obvious differentiation in flower morphologies between the bisexual flowers and the female/male mutants. The female flowers had smaller columns with degenerated stamens without the pollinia, narrower elliptic sepals, labellate and curling downward petals, and apiculate ovate labella (Figure 1(B1,B2)). The male flowers had round obovate sepals and petals, short and stout columns, degenerated pistils and stigma cavities, and short retreated ovaries (Figure 1(C1,C2)). The ovary of the male (11.73 ± 0.21 mm) flower is degenerated and shorter than that of the female (20.05 ± 1.17 mm) and hermaphrodite bisexual (19.14 ± 0.12 mm) flowers (*t*-test, both *p* < 0.05; Table S1; Figure 1A–C). 

### 2.2. Transcriptome Sequencing, Assembly, and Annotation

The gynostemium and the ovary, which are the key sex organs determining the sexual phenotypes in *C. tortisepalum*, were selected as materials for comparative transcriptomes. Mixed samples (COOV) of the gynostemium column and ovary for three sexes (F-COOV, H-COOV, and M-COOV, Figure 1(A2–C2)) were collected with three replicates for RNA sequencing. A total of 85.71 Gb of clean transcriptome data were generated for nine COOV samples after quality control (Table S2), with the error rate < 0.025%, the GC content between 45.58 and 47.97%, and the Q30 base percentage > 93.83%. A total of 455,213 transcripts were assembled from the transcriptome data (Table S3). In total, 320,413 unigenes were identified from those transcripts. A total of 2056 transcripts had sequence lengths > 4500 bp (0.45% of the total transcripts), while 1313 unigenes had lengths > 4500 bp (0.41% of the total unigenes) (Table S4). BUSCO assignments showed that the N50 was 1067 bp, the GC content was 38.23%, and the assembly had 56.9% completeness (Table S3).

In total, 181,470 (39.86%) transcripts and 106,704 (33.3%) unigenes were successfully annotated functionally (Table S5). For the transcripts, the libraries of NR, Swiss-Prot, Pfam, COG, GO, and KEGG obtained 169,522 (37.24%), 107,880 (23.7%), 92,183 (20.25), 20,483 (4.5%), 106,269 (23.34%), and 70,382 (15.46%) annotations, respectively (Table S5). In addition, the NR annotation hit 26.31%, 8.22%, and 65% of unigenes in *Dendrobium catenatum*, *Phalaenopsis equestris,* and other species homologs, respectively (Table S6). In the transcripts, a total of 3421 genes were predicted to be transcription factors belonging to 35 gene families (Figure S1). Among these, the MYB superfamily and the C2C2, bHLH, bZIP, AP2/ERF, and NAC families had 612, 264, 229, 227, 222, and 213 genes, respectively, accounting for 51.65% (1767 genes) of the total transcripts. In the unigenes, a total of 34 transcription factor gene families were predicted. A total of 1563 genes, including the MYB, C2C2, AP2/ERF, bZIP, C3H, and NAC families, which had 257, 122, 122, 111, 96, and 94 genes, respectively, accounted for 51.31% (802 genes) of the total unigenes.

### 2.3. Expression Levels among the Three Sexual Phenotypes

In total, 46,719 genes were expressed in the female COOV samples, 45,678 in the male COOV samples, and 50,555 in the hermaphroditic COOV samples (Figure S2A). A principal component analysis (PCA) of the expression levels in the COOV samples of the three sexual phenotypes (F-COOV, H-COOV, and M-COOV) showed that the expression levels of unigenes among them were different. The expression levels in the COOV samples for each sexual phenotype were clustered together within each sexual phenotype except for samples H_lb_coov3 and F_sxd_coov3 (Figure S2B). The distances between the unigenes expressed in the COOV samples among the sexual phenotypes were relatively far, which indicates a large difference in the expression levels among them. However, there was some difference in the expression level among the samples within each sexual phenotype, which might be the result of the different genetic backgrounds among the plant materials within the same sexual phenotype (Figure S2B). A correlation analysis of gene expression showed low similarity among the three sexual phenotypes but small difference within each sexual phenotype, indicating a significant difference in the expression levels among the female, male, and hermaphroditic plants (Figure S2C). Among them, 12,145, 10,789, and 14,447 genes were uniquely expressed in the female, male, and hermaphrodite COOV samples, respectively, while 28,557 genes were common (Figure S2D).

### 2.4. Sex-Biased DEGs among the Three Sexual Phenotypes

**Figure 2:**
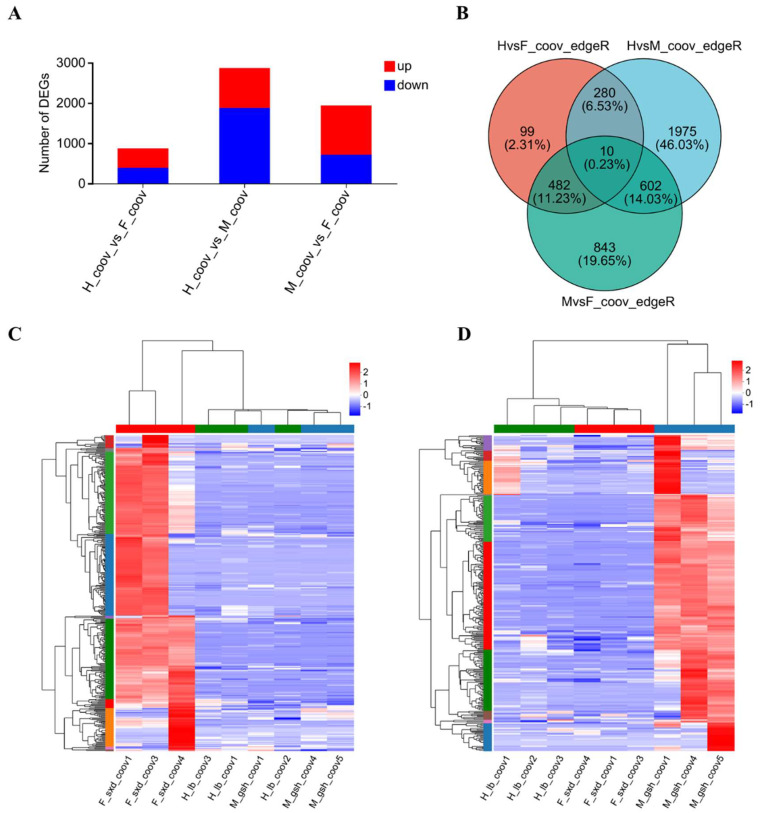
Expression differentiation for the COOV (mix of gynostemium and ovary) samples among three sexual phenotypes. (**A**) Statistical map of 4291 deduplicated sex-biased DEGs for three pairwise comparisons (H-COOV vs. F-COOV, H-COOV vs. M-COOV, M-COOV vs. F-COOV) of COOV samples among hermaphrodite, female, and male sexual phenotypes. (**B**) Venn diagram of DEGs for three pairwise comparisons. (**C**) Cluster heat map of DEGs of COOV samples showing upregulated intersected DEGs in the comparison of female samples with hermaphrodite and male samples. (**D**) Cluster heat map of DEGs of COOV samples showing upregulated intersected DEGs in the comparison of male samples to hermaphrodite and female samples.

To investigate the expression differences in the COOV samples among the three sexual phenotypes in *C. tortisepalum*, differentially expressed genes (DEGs) were identified. Through a pairwise comparisons of the three groups (H-COOV vs. F-COOV, H-COOV vs. M-COOV, M-COOV vs. F-COOV) for the expression levels of the COOV samples among the three sexual phenotypes, we identified a total of 4291 deduplicated sex-biased DEGs, with 871 DEGs (483 upregulated and 388 downregulated) between the H-COOV and F-COOV samples, 2867 DEGs (990 upregulated and 1877 downregulated) between the H-COOV and M-COOV samples, and 1937 DEGs (1223 upregulated and 714 downregulated) between the M-COOV and F-COOV samples (Figure 2A–D and Figure S3). The Venn diagram and Volcano plot showed only 10 intersected DEGs among the three groups of the pairwise comparisons, 290 intersected DEGs between the comparisons of the H-COOV vs. F-COOV and H-COOV vs. M-COOV samples, 612 intersected DEGs between the comparisons of the H-COOV vs. M-COOV and M-COOV vs. F-COOV samples, and 492 intersected DEGs between the comparisons of the H-COOV vs. F-COOV and M-COOV vs. F-COOV samples (Figure 2B).

### 2.5. GO and KEGG Enrichments of Sex-Biased DEGs

The 871 DEGs in the H-COOV vs. F-COOV comparison were mainly enriched in the GO terms of xanthoxin dehydrogenase activity, symbiont process, alcohol dehydrogenase (NAD) activity, and lipid glycosylation and transport (Figure S4A), and in the KEGG pathways of phenylpropanoid biosynthesis, isoquinoline alkaloid biosynthesis, alpha-linolenic acid metabolism, and MAPK signaling (Figure S4D). The 2867 DEGs between the H-COOV and M-COOV samples were enriched in the GO terms of xyloglucan metabolic process, hydrolase activity, hydrolyzing O-glycosyl compounds, and polysaccharide metabolic process (Figure S4B), and in the KEGG pathways of phenylpropanoid biosynthesis, flavonoid and sesquiterpenoids/terpenoids biosynthesis, DNA replication, and starch and sucrose metabolism (Figure S4E). The 1937 DEGs in the comparison of the M-COOV vs. F-COOV samples were mainly enriched in the GO terms of extracellular region, protein binding, hydrolase activity, microtubule-based movement, and xyloglucan metabolic process (Figure S4C), and in the KEGG pathways of phenylpropanoid biosynthesis, sesquiterpenoid/triterpenoid biosynthesis, plant hormone signal transduction, linoleic acid metabolism, and DNA replication (Figure S4F).

For the 492 intersecting DEGs between the comparisons of the H-COOV vs. F-COOV and M-COOV vs. F-COOV samples, 369 upregulated genes in the female COOV samples were mainly enriched in the GO terms of lipid glycosylation, xanthoxin dehydrogenase activity, acetylglucosaminyltransferase activity, microtubule binding, and RNA–DNA hybrid ribonuclease activity (Figure S5A), and in the KEGG pathways of stilbenoid, diarylheptanoid and gingerol biosynthesis, glycerophospholipid metabolism, ether lipid metabolism, and ABC transporters (Figure S5C). For the 612 intersecting DEGs between the comparisons of the H-COOV vs. M-COOV and M-COOV vs. F-COOV samples, 315 upregulated genes in the male COOV samples were mainly enriched in the GO terms of L-leucine transaminase activity, branched-chain-amino-acid transaminase activity, serine-type endopeptidase activity, regulation of homotypic cell–cell adhesion, and microtubule-based process (Figure S5B), and in the KEGG pathways of cysteine and methionine metabolism, stilbenoid, diarylheptanoid and gingerol biosynthesis, as well flavonoid biosynthesis (Figure S5D).

### 2.6. Co-Expression Networks of Sex-Biased DEGs

**Figure 3:**
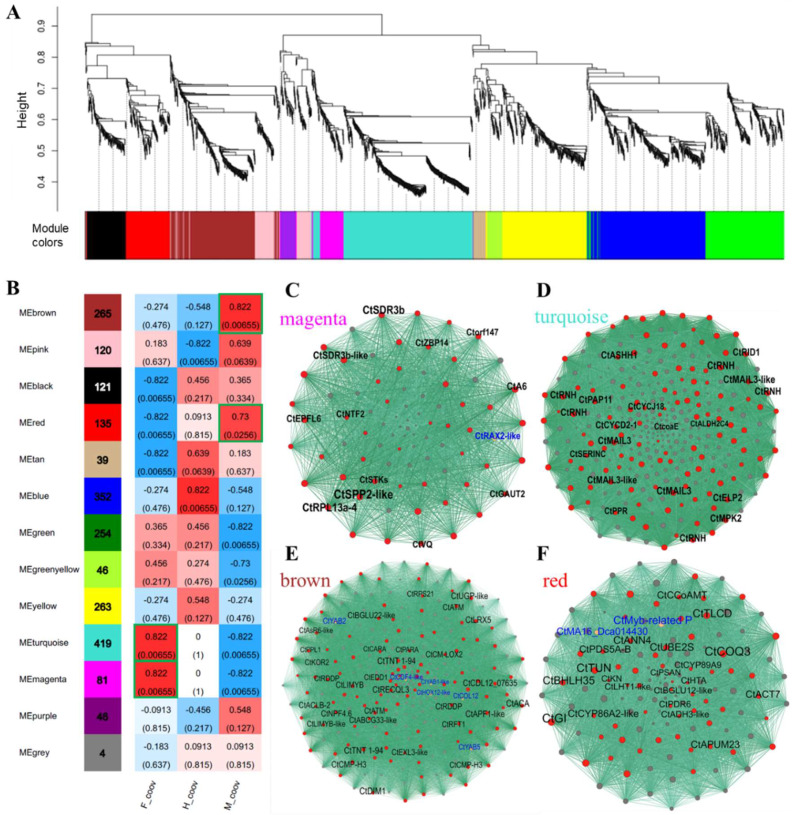
Co-expression networks of 4291 sex-biased DEGs in pairwise comparisons (H-COOV vs. F-COOV, H-COOV vs. M-COOV, M-COOV vs. F-COOV) of the COOV (mix of gynostemium and ovary) samples among three sexual phenotypes (female, male, and hermaphrodite flowers). (**A**) Gene dendrogram and module clusters of WGCNA analysis. (**B**) Thirteen modules clustered in WGCNA for 4291 sex-biased DEGs among the COOV samples of female (F-COOV), male (M-COOV), and hermaphrodite (H-COOV) flowers. The top two positive-related modules were marked by green frames for F-COOV and M-COOV traits. (**C**) Co-expression network of female module MEmagentia. *CtSDR3b*, *CtSDR3B-like, CtEPFL6,* and *CtA6* (*glucan endo-1,3-beta-glucosidase A6*) genes as well as one *CtRAX2-like* transcriptional factor were nested in this module. (Blue highlighted genes are transcriptional factors, similarly hereinafter) (**D**) Co-expression network of female module MEturquoise. (**E**) Co-expression network of male module MEbrown. Transcriptional factors *CtYAB2* and *CtYAB5*, as well as *CtYAB1-like*, *CtCDF4-like*, *CtHOX12-like, and CtCOL-12* genes were nested in this module. (**F**) Co-expression network of male module MEred.

To further determine the potential core gene networks responsible for the sex differentiation of *C. tortisepalum*, a co-expression network was established by applying WGCNA analysis (Figure 3A) for 4291 deduplicated sex-biased DEGs of the pairwise comparisons (H-COOV vs. F-COOV, H-COOV vs. M-COOV, M-COOV vs. F-COOV) among the three sexual phenotypes. These 4291 sex-biased DEGs were clustered into thirteen modules with five modules positively and tightly correlated with sexual traits (*R* > 0.7, *p* < 0.05; Figure 3B). 

Two modules, MEmagenta (81 genes, *R* = 0.822, *p* = 0.0066) and MEturquoise (419 genes, *R* = 0.822, *p* = 0.0066), were highly correlated with the female traits (Figure 3B). There were 49 hub genes in the co-expression network of female module MEmagenta, including the *CtSDR3b* (*short-chain dehydrogenase reductase 3b*)*, CtSDR3b-like*, *CtEPFL1* (*Epidermal patterning factor/EPF-like 1*) and *CtA6* (*glucan endo-1,3-beta-glucosidase A6* genes), as well as a transcription factor *CtRAX2-like* (Figure 3C; Table S7). The *CtSDR3b* (*TRINITY_DN154464_c1_g1*) and *CtSDR3b-like* (*TRINITY_DN162927_c2_g1*) genes as the hub genes of female module MEmagenta were also the top significant female-biased candidate DEGs (Figure 3C; Table S7) and were directly connected to the genes enriched in the GO terms of xanthoxin dehydrogenase activity, alcohol dehydrogenase (NAD) activity, sugar-phosphatase activity, and mitochondrion (Table S8). Moreover, there were 122 hub genes in the co-expression network of female module MEturquoise, including two *CtMAIL3* genes, two *CtMAIL3-like* genes and the *CtRID1* gene (Figure 3D; Table S7). The genes in module MEturquoise were enriched in tubulin binding, lipid glycosylation, acetylglucosaminyltransferase activity, and aldehyde dehydrogenase [NAD(P)+] activity, which might also participate in the sex differentiation of the female flowers (Table S8).

Two modules, MEbrown (265 genes, *R* = 0.822, *p* = 0.0066) and MEred (135 genes, *R* = 0.73, *p* = 0.0256), were highly correlated with the male traits (Figure 3B). There were 126 hub genes in the co-expression network of male module MEbrown, including *CtYAB5, CtYAB2, CtLIMYB* and *CtABCG33-like* (Figure 3E; Table S7). Significantly male-biased expressed candidate DEGs, *CtYAB5* (*TRINITY_DN162642_c1_g2*) and *CtYAB2* (*TRINITY_DN173328_c4_g2*), as the hub genes, were nested in the co-expression network of male module MEbrown (Figure 3E; Table S7) and were directly connected to the genes enriched in the GO terms of multicellular organism development, phenylpropanoid catabolic process, polygalacturonase activity, and anatomical structure development (Table S8). In addition, there were 62 hub genes in the co-expression network of male module MEred, including *CtbHLH35*, *CtGIGANTEA* (*GI*), and *CtADH3-like* (Figure 3F; Table S7). The genes in the co-expression network of the MEred module were enriched in the GO terms of pigment metabolic process, movement of cell or subcellular component, regulation of reproductive process, and response to UV, which might also participate in the sex differentiation of the male flowers (Table S8).

### 2.7. Candidate Genes of Sex Differentiation and Their Expression Patterns

**Figure 4:**
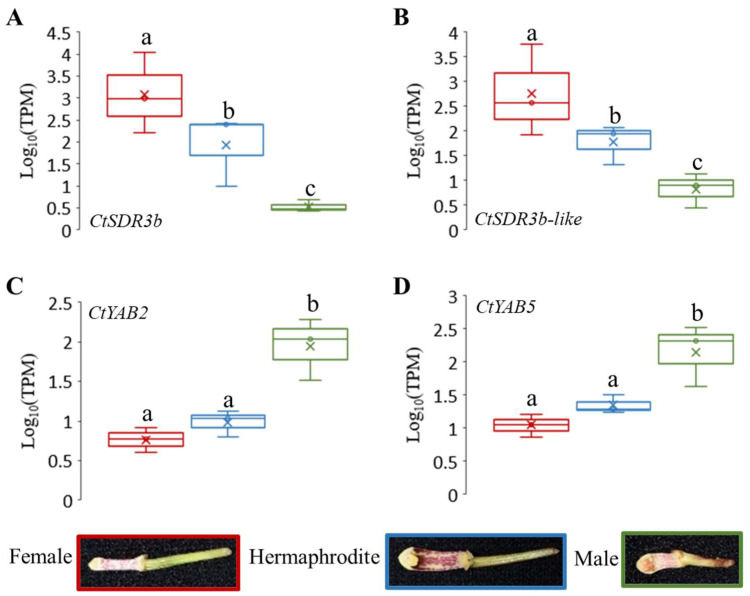
Expression patterns of four candidate genes of sex differentiation in COOV (mix of gynostemium and ovary) samples for different sexual phenotypes in *C. tortisepalum*. The expression levels are shown by the statistics log_10_(TPM), which was transformed from the expression TPM values for the candidate genes *CtSDR3b* (**A**), *CtSDR3b-like* (**B**), *CtYAB2* (**C**), and *CtYAB5* (**D**). The letters above each bar show the difference significance level in DEG analysis between sexual groups in edgeR. The same letter above the error bars indicates adjusted *p* > 0.05, while different letters indicate adjusted *p* < 0.05.

Among the 369 upregulated intersected DEGs in the female COOV samples compared to the hermaphrodite and male COOV samples, as well as the 315 upregulated intersected DEGs in the male COOV samples compared to the hermaphrodite and female COOV samples, the most significant DEGs that potentially participate in pistil/stamen development and sex differentiation were annotated and identified (Figure 2C,D). Based on the evidence of the sex-biased top DEGs as well as the hub genes of the sex-related modules identified in the co-expression networks (Figure 2C,D and Figure 3), the *CtSDR3b* and *CtSDR3b-like* genes, which had the highest expression in the female COOV samples and the lowest expression in the male and bisexual COOV samples, were selected. In addition, the *CtYAB5* and *CtYAB2* genes with low expression in the female COOV samples and high expression in the male and bisexual COOV samples were identified (Figure 4). The *CtSDR3b* and *CtSDR3b-like* genes are the homologs of the *SDR3b* and *SDR3b-like* genes of maize sex determination factor *tasselseed2* [32], while *CtYAB5* and *CtYAB2* are the homologs of transcription factors *YABBY5* and *YABBY2* of grape sex determination factor *YABBY3* [33]. Based on the above evidence of the sex-biased top DEGs, the hub genes of the co-expression networks in the female and male modules, as well as the previous studies of sex determination/differentiation [32,33], we proposed that these four candidate genes might play critical roles in sex differentiation in *C. tortisepalum*.

The relative expression of *CtSDR3b* was significantly upregulated in the female COOV samples (log_10_TPM = 3.08 ± 0.926 (mean ± s.e.); Figure 4A) but downregulated in the male and hermaphrodite COOV samples (male log_10_TPM = 0.529 ± 0.132; hermaphrodite log_10_TPM = 1.936 ± 0.807; adjusted *p* < 0.05). The expression pattern of *CtSDR3b-like* was similar to that of *CtSDR3b* (male log_10_TPM = 0.825 ± 0.351; hermaphrodite log_10_TPM = 1.779 ± 0.404; female log_10_TPM = 2.748 ± 0.933, Figure 4B). The relative expression of *CtYAB2* was significantly upregulated in the male COOV samples (log_10_TPM = 1.947 ± 0.389) but significantly downregulated in the female and hermaphrodite COOV samples (female log_10_TPM = 0.764 ± 0.161; hermaphrodite log_10_TPM = 0.987 ± 0.166; adjusted *p* < 0.05; Figure 4C). The expression pattern of *CtYAB5* was similar to that of *CtYAB2* (male log_10_TPM = 2.151 ± 0.468; hermaphrodite log_10_TPM = 1.346 ± 0.138; female log_10_TPM = 1.045 ± 0.167; Figure 4D). According to the expression patterns of the four candidate genes among the three sexual phenotypes, the *CtSDR3b* and *CtSDR3b-like* genes might have a potential role in determining the female flower sex organ, while the *CtYAB5* and *CtYAB2* genes might have a potential role in determining the male flower sex organ.

### 2.8. qRT-PCR Validation of Sex Differentiation Candidate Genes

**Figure 5:**
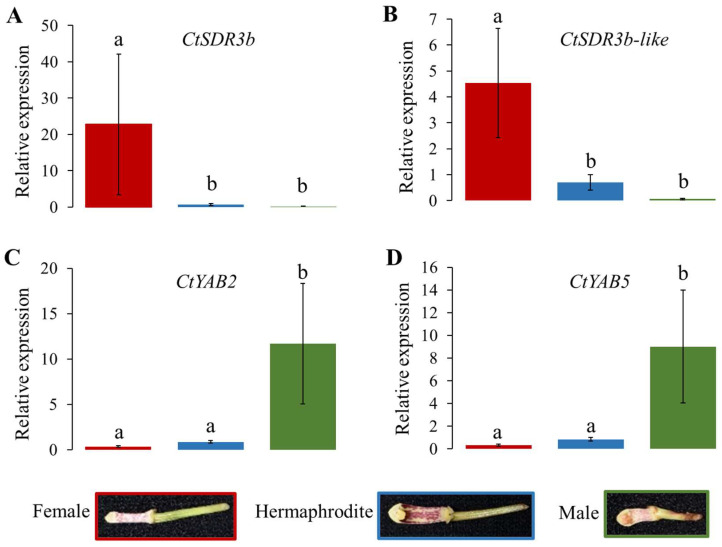
qRT-PCR results for four candidate sex differentiation genes in the COOV (mix of gynostemium and ovary) samples for three *C. tortisepalum* sexual phenotypes. (**A**) *CtSDR3b*, (**B**) *CtSDR3b-like*, (**C**) *CtYAB2*, and (**D**) *CtYAB5*. The letters above each bar show the difference significance level of *Z*-*test* for log_2_(x+1) transformation of expression data and log_2_FC values between sexual groups. The same letter above the error bars indicates either log_2_FC < 1.5 or *p* > 0.05, while different letters indicate both log_2_FC > 1.5 and *p* < 0.05.

qRT-PCR experiments were performed to verify the expression of four candidate genes in the COOV samples of *C. tortisepalum* using the designed primers (Table S9). The expression patterns of the four sex-determining candidate genes validated in the qRT-PCR were consistent with those shown in the RNA-seq results (Figure 4 and Figure 5). The average expression level of the *CtSDR3b* gene was significantly upregulated in the female COOV samples (22.764 ± 19.37) compared to the male (0.186 ± 0.083) and hermaphrodite (0.708 ± 0.292) samples (Figure 5A; both log_2_FC > 1.5 and *Z*-test, *p* < 0.05). Similarly, the average expression level of the *CtSDR3b-like* gene was significantly upregulated in the female COOV samples (4.541 ± 2.111) compared to the male (0.061 ± 0.022) and hermaphrodite (0.701 ± 0.299) samples (Figure 5B; both log_2_FC > 1.5 and *Z*-test, *p* < 0.05). Conversely, the average expression level of the *CtYAB2* gene was significantly upregulated in the male COOV samples compared to that of the female and hermaphrodite COOV samples (Figure 5C; male = 11.688 ± 6.651; female= 0.355 ± 0.079; hermaphrodite = 0.862 ± 0.138; both log_2_FC > 1.5 and *Z*-test, *p* < 0.05). A similar expression pattern of the *CtYAB5* gene was also detected, with the upregulated average expression level in the male COOV samples (9.011 ± 4.976) compared to that in the female (0.342 ± 0.084) and hermaphrodite (0.842 ± 0.158) samples (Figure 5D; both log_2_FC > 1.5 and *Z*-test, *p* < 0.05).

## 3. Discussion

Although lots of studies have investigated the pollination strategies and reproductive organ specification in the orchid family, few studies have explored their sexual system and the underlying molecular mechanism. In our *C. tortisepalum* study, phenotypic measurements showed that the abnormal sexual organs in the female and male plants are different from that in the normal hermaphrodite plants, which was caused by the corresponding mutations of the gynostemium, pollinia, stigma cavity, and ovary (Figure 1). Furthermore, based on the comparative transcriptomes, the sex-specific expression profiles as well as co-expression networks of the flower sex organs (gynostemium and ovary), our results revealed a total of 4291 sex-biased DEGs in the sex organs among the female, male, and hermaphrodite *C. tortisepalum* flowers. Two co-expressed network modules with 81 and 419 genes, respectively, were highly correlated with the female sexual traits, while the two other modules with 265 and 135 genes were tightly correlated with the male sexual traits. Based on these results, we constructed the sex-related gene networks for the orchid *C. tortisepalum*.

It was proposed that in the female-related gene network, several hub genes nested in the two WGCNA modules may participate in the sex differentiation of the *C. tortisepalum* female flower. The *SDR3b* and *SDR3b-like* hub genes, nested in female module MEmagentia, are the members of the SDR superfamily that appears to be restricted to monocots [34]. They encode oligopeptides containing 250 to 300 amino acid residues and are mainly NAD- or NADP-dependent REDOX enzymes. The *SDR3b* and *SDR3b-like* genes are homologous to *tasselseed2,* which is the sex determination factor of *Zea mays* [32,35]. This gene encodes short peptides that regulate and initiate the programmed cell death of the female organ primordia during the flower development process [36], which is critical for sex determination/differentiation in *Zea mays,* and even in Poaceae [34]. In maize *tassel seed* mutants, tassel sex expression in both recessive mutants (*tasselseed1*, *tasselseed2*), as well as dominant mutants (*Tasselseed3*, *Tasselseed5*), is reversed with the gynoecium developed and stamens ectopically suppressed [32,37]. The loss-of-function mutation of *tasselseed2* causes a complete feminization of all tassel florets, while the overexpression of *Tasselseed5* leads to the formation of feminized tassels by affecting jasmonate catabolism [32,37,38]. Based on the previous studies as well as the expression patterns of the *CtSDR3b* and *CtSDR3b-like* genes, which are the top female-biased DEGs among the three sexual phenotypes (Figure 4 and Figure 5), we proposed that the *CtSDR3b* and *CtSDR3b-like* genes in the female flowers might functionally abort the programmed cell death (PCD) of the female primordium then promote flower organ feminization in *C. tortisepalum*. The upregulation of these genes might lead to the *C. tortisepalum* flower feminization with the absence of the pollinia during the development of the *C. tortisepalum* flower (Figure 1 and Figure 4). Moreover, one hub gene in female module MEmagentia, the *CtEPFL6*, is a homolog of the wheat *EPIDERMAL PATTERNING FACTOR-LIKE* (*EPFL*) secreted peptide gene that plays an important role in stamen development. The overexpression of *EPFL1* results in abnormal stamens in wheat [39]. In *Arabidopsis*, the EPFL peptide ligands which are expressed in the epidermal layer, together with the ERf receptor kinases, control the female germline specification [40]. The other hub gene in the MEmagentia module, *CtA6* (*glucan endo-1,3-beta-glucosidase A6*), is the homolog of the gene associated with the cytoplasmic male sterility of *Gossypium harknessii* [41]. The upregulation of these two hub genes in the *C. tortisepalum* female flower might also be responsible for female sex differentiation via male sterility or female specification. 

It was proposed that in the male-related gene network, several hub genes including few transcriptional factors nested in the two WGCNA modules may participate in the *C. tortisepalum* male sex differentiation. Both *CtYAB5* and *CtYAB2*, nested in male module MEbrown, belong to the YABBY transcription factor family restricted to the plant kingdom’s members that contain two conserved structural domains: the zinc finger domain (N-terminal) and the YABBY domain (C-terminal) [42]. The members of the YABBY family are involved in plant development, specifically, in the adaxial–abaxial polarity differentiation of lateral organs [42,43]. Recent studies have shown that the YABBY family plays an important regulatory role in the development of flower organs [44,45,46,47]. The potential female sterility function was also revealed to be associated with the *YABBY3*, which acts as a sex determination factor in grapes [33]. Two male-linked nonsynonymous SNPs in the *YABBY3* gene represent potential female-sterility mutations in grapes [33]. In our study, both the RNA-seq and qRT-PCR indicated that the expression of the *CtYAB5* and *CtYAB2* genes was significantly upregulated in the male COOV samples compared to the female and hermaphrodite plants (Figure 4 and Figure 5). A previous study of a *Phalaenopsis* orchid showed that transient overexpression of the *PeDLs* genes, the members of the DROOPING LEAF/CRABSCLAW (DL/CRC) subfamily within the YABBY gene family, caused abnormal development of the ovule and the stigmatic cavity of the gynostemium in *Phalaenopsis* [47]. We hypothesized that the *CtYAB5* and *CtYAB2* genes in *C. tortisepalum* might have similar functions to those of the *YABBY3* gene in grapes as well as of the *PeDL* genes in *Phalaenopsis*, whose mutation may cause female sterility during the orchid flower development, thus forming the male sexual traits of *C. tortisepalum*. The upregulation of these genes might lead to the *C. tortisepalum* flower androphany with the absence of the ovary or the degeneration of the stigma cavity during the development of the *C. tortisepalum* flower (Figure 1 and Figure 4). 

**Figure 6:**
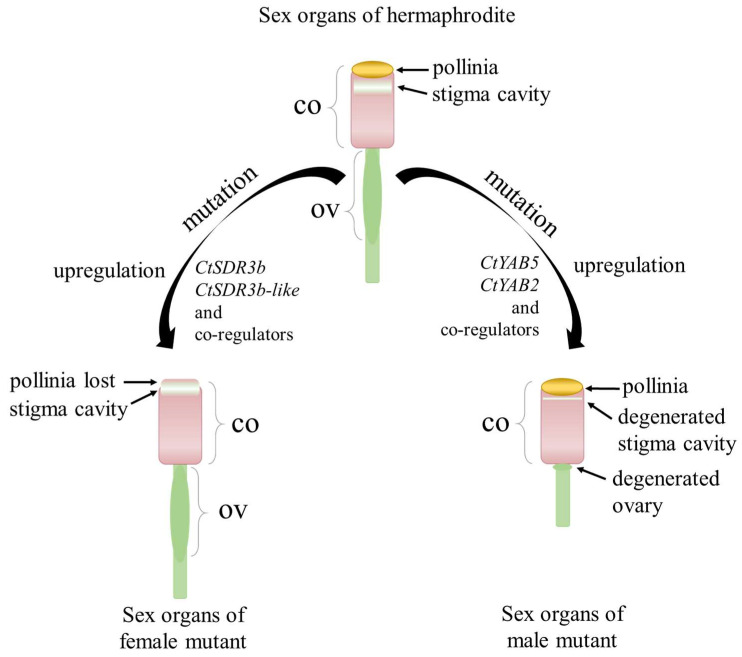
Proposed model of sex determination/differentiation mechanism in *C. tortisepalum*. The model shows the sex separation of the flower sex organs including the gynostemium (column, CO) and ovary (OV).

Based on our comparative transcriptome studies of the sex-related gene networks, we proposed a regulation model of sex differentiation for the orchid *C. tortisepalum* (Figure 6). Our data provide some evidence for such model, in which the upregulated expression of the *CtSDR3b* and *CtSDR3b-like* genes and other co-regulators might co-regulate the feminization of *C. tortisepalum* with the absence of the pollinia during flower development that forms the female plants. Meanwhile, the upregulated expression of the transcription factor *CtYAB2* and *CtYAB5* genes and other co-regulators might co-regulate the androphany of *C. tortisepalum* with the absence of the ovary and the degeneration of the stigma cavity during flower development that forms the male plants. 

## 4. Materials and Methods

### 4.1. Morphological Measurements of Three Sexual Phenotypes

The whole hermaphrodite (H) plants, as well as female (F) and male (M) mutants of *C. tortisepalum* were collected by the cloning of the initial individuals of natural population in 2012 from the mountain area of Baoshan City, Yunnan Province, Southwest China. Three sexual types have the abilities to intercross each other [48]. They were collected and planted in the Orchid Garden of Fujian Agriculture and Forestry University. To compare the three sexual phenotypes, the height of each plant was measured. Moreover, the flower was dissected into the sepals, petals, labellum, gynostemium (column), and ovary. The length and width of each flower part were measured by digital Vernier calipers with three replicates. The average value of each measurement was calculated for each sexual phenotype. The plants and flower parts were photographed using a Nikon/Nikon D 810 camera.

### 4.2. RNA Sample Collection and Extraction

The male, female, and hermaphrodite plants of *C. tortisepalum* were planted in the Orchid Garden of Fujian Agriculture and Forestry University. The flower column (gynostemium) and ovary of each sexual phenotype were collected and mixed as COOV (column + ovary; Figure 1(A2–C2)) samples for RNA extraction. Samples were repeated three times from three individual plants (three flowers per each individual were pooled as one sample) for each sex and frozen in liquid nitrogen then immediately placed at −80 °C for subsequent RNA extraction. Total RNA was extracted from each COOV sample using TRIzol (Invitrogen, Carlsbad, CA, USA). RNA concentration was measured using a Nanodrop 2000 (Thermo Fisher Scientific, Waltham, MA, USA). The RNA quality was evaluated by the ratio of OD 260/280 (1.8–2.0) and the ratio of OD 260/230 (1.9–2.2). The RNA integrity was determined by 1% agarose electrophoresis. RNA samples without obvious protein, sugar, or other pollution met the requirements for constructing the cDNA library.

### 4.3. Construction of cDNA Library and Transcriptome Sequencing

After obtaining high-quality RNA samples, a TruSeq TM RNA sample preparation kit (Illumina, San Diego, CA, USA) was used to construct a library. First, magnetic beads with oligo (dT) from 10 µg of total RNA were used to enrich mRNA. Second, fragmentation buffer was added to fragment mRNA into 200 bp small fragments. Third, a SuperScript double-stranded cDNA synthesis kit (Invitrogen, CA, USA) was used to synthesize the first chain of cDNA, and then the solution and RNase H were added to synthesize the two chains to form a stable double-stranded structure. After PCR enrichment, a 2% agarose gel was used for gel electrophoresis to select 250-300 bp DNA fragments. The fragment was then used to construct the PE 150 bp library, while the raw reads were generated by RNA sequencing using the Illumina Novaseq 6000 platform (Illumina, San Diego, CA, USA). To obtain the high-quality clean data for subsequent analysis, the SeqPrep (https://github.com/jstjohn/SeqPrep (accessed on: 10 January 2020)) and Sickle (https://github.com/najoshi/sickle (accessed on: 10 January 2020)) were used for quality control. The specific steps are as follows: (1) remove the adaptor sequence in reads; (2) prune away the base at the end of the column (3′ end) with low quality (≤20); (3) remove the reads with rates higher than 10% N base; (4) discard the short sequence (≤30 bp) after removing the adapter. 

### 4.4. De Novo Transcriptome Assembly

High-quality clean reads were used for transcript assembly by Trinity v2.8 [49]. The assembly process was as follows: First, Inchworm reads were decomposed, a k-mer (default K = 25) dictionary was constructed, and the seed k-mer was extended on both sides to form contigs. Second, Chrysalis: Overlapped contigs were used to construct the components. Each component became a collection of representations of variable-splice isoforms or homologs and had a compatible de Bruijn graph. Third, Butterfly: The de Bruijn graph was simplified to output the full-length transcript of the variable shear subtype and combine the transcript of paracyclic homologous gene. Finally, the spliced results were obtained.

TransRate v1.0.3 was used to evaluate common errors in the assembly results, and contigs were scored successively [50]. At this stage, the composite score of the whole assembly result can be obtained. In the case of redundant sequences and high similarity, CD-HIT was used to eliminate redundancy [51]. Redundant and similar sequences were removed by alignment to the NR database. Finally, BUSCO v4. software was used to assess the integrity of the assembly results [52].

### 4.5. Functional Annotations and Transcription Factor Prediction

Blastx was used to compare unigene sequences with NR (ftp://ftp.ncbi.nih.gov/blast/db/ (accessed on: 10 January 2020)), Pfam (http://pfam.xfam.org/ (accessed on: 10 January 2020)), KOG/COG/EGGNOG (ftp://ftp.ncbi.nih.gov/pub/COG/COG (accessed on: 10 January 2020); http://eggnogdb.embl.de/ (accessed on: 10 January 2020)), Swiss-Prot (http://www.uniprot.org/ (accessed on: 10 January 2020)), KEGG (http://www.genome.jp/kegg/ (accessed on: 10 January 2020)), and GO (http://www.geneontology.org/ (accessed on: 10 January 2020)). KOBAS2.0 was used to analyze the results of KEGG orthology of unigenes [53]. HMMER v3.4 [54] was used to predict the annotation information for the unigenes with the Pfam database and to compare the unigenes with the PlantTFDB database (http://planttfdb.cbi.pku.edu.cn/ (accessed on: 10 January 2020)) to obtain the information on the transcription factors and their families for *C. tortisepalum* [55].

### 4.6. Identification of Differentially Expressed Genes (DEGs)

The high-quality reads were compared to the Unigene library using Bowtie v1.3.0 [56]. According to the comparison results, RSEM v1.3.2 [57] was used to estimate the expression level. The TPM (transcripts per million) value was proven to reflect the overabundance of unigenes. TPM is calculated as follows:

In the formula, X and L represent gene read counts and gene length, respectively.

$${\mathrm{TPM}}_{i}=\frac{X_{i}}{\tilde{l_{i}}}\cdot \left(\frac{1}{\sum_{j}\frac{X_{j}}{\tilde{l_{j}}}}\right)\cdot 10^{6}$$

The R package edgeR [58] was used to account for the read counts of COOV samples of different sexual phenotypes in *C. tortisepalum* flowers to obtain the differentially expressed unigenes/transcripts between different sexual phenotypes as the differentially expressed genes (DEGs). The difference significance level of DEG analysis was calculated in edgeR with the *p*-value adjusted by Bonferroni correction.

### 4.7. GO and KEGG Enrichment of DEGs

GO functional enrichment and KEGG pathway enrichment analysis were performed on the differentially expressed genes (DEGs) obtained in the previous step. The DEGs related to the formation of different sexes were screened. GO function enrichments and KEGG pathway enrichments of genes/transcripts were performed using the R package of ClusterProfile with Fisher precise tests [59]. To prevent the false positives of significant tests, the Bonferroni correction was used to correct the *p*-value for the enrichment analysis.

### 4.8. Construction of the Co-Expression Network

The DEGs in the datasets were selected individually and subjected to the R package WGCNA [60]. WGCNA network construction and module detection were conducted using an unsigned type of topological overlap matrix (TOM) with the following parameters: soft power = 5, minModuleSize = 30 and mergeCutHeight = 0.25. Genes in the male module of stage 1 (MS1) related to the formation of male organs directly linked with candidate sex determinants were visualized using the VisANT v5.51 program [61]. The final network was designed using the igraph package [62].

### 4.9. qRT-PCR Validation

The expression patterns of candidate genes in this study were validated by qRT-PCR. The tissues of gynostemium (column) and ovary were collected at the flower bud stage then mixed as the COOV samples for three sexes (with female three replicates, male five replicates, while hermaphrodite three replicates). The samples were frozen in liquid nitrogen and then placed at −80 °C for RNA extraction. The stable expressed *Actin* gene (accession No. GU181353) from congener species was chosen as the reference gene [63]. The primers for the reference gene and four candidate genes were designed using Primer Premier v5.0 [64].

The Applied Biosystems^®^ QuantStudio^®^3 real-time fluorescence quantitative PCR system (Applied Biosystems, Foster City, CA, USA) was used with the SYBR Green dye method for 20 μL reaction: 2 μL cDNA, 0.8 μL upstream and downstream primers and dye, 10 μL SYBR Premix ExTaq TM (TaKaRa), 5.6 μL ddH_2_O, three technical replicates for each sample. The reaction program was set as follows: pre-denaturation at 93 °C for 2 min; denaturation at 93 °C for 51 min, annealing at 55 °C for 2 min; 40 cycles. The signal of fluorescence was collected at the end of the 55 °C step. The product specificity was determined by the melting curve: 55 °C was slowly raised to 96 °C. The *Ct* value of each sample was obtained, and the expression stability of reference gene was evaluated using NormFinder [65,66,67]. The relative expression of the target genes was quantitatively calculated through the 2^−ΔΔCt^ method [68]. Log_2_Foldchange of relative expression values were calculated and *Z*-*test* were performed to determine the differences in expression level among three sexes.

## 5. Conclusions

Our study was the first to discover the sex-related gene networks that contribute to the sex differentiation in the orchid *Cymbidium tortisepalum* and proposed a potential pathway that leads to the separation of sex organs in the orchid family. The *C. tortisepalum* unisexual mutants have obviously degenerated phenotypes compared to the bisexual plants not only in the flower sexual reproduction organs but also in the whole plant morphologies. Two co-expressed modules were highly correlated with the feminized sexual traits of the *C. tortisepalum* flower potentially determined by two candidate hub genes (*CtSDR3b* and *CtSDR3b-like*), while the two other modules highly correlated with the androphany sexual traits possibly determined by the two other candidate hub genes (*CtYAB2* and *CtYAB5*). Because sex determination systems are not conserved among different lineages in plants [3,4], the heterologous genetic transformation using model plants such as rice or *Arabidopsis* may not be sufficient to validate the candidate genes for the orchid *C. tortisepalum*. However, further development of a stable genetic transformation system for the *Cymbidium* or Orchidaceae plants will provide a chance for more sufficient validation of the candidate *C. tortisepalum* sex differentiation genes in future studies [69,70]. Nonetheless, our study provided the experimental evidence of and proposed the genomic/genetic basis for sex differentiation and reproductive organ innovation in the orchid family.

## Data Availability

The Illumina reads of transcriptome sequences have been submitted to the Genome Sequence Archive (GSA), National Genomics Data Center (NGDC), under BioProject PRJCA020531.

## Supplementary Materials

The following supporting information can be downloaded at https://www.mdpi.com/article/10.3390/ijms242316627/s1.
