# Supplementary material for: Sex-Related Gene Network Revealed by Transcriptome Differentiation of Bisexual and Unisexual Flowers of Orchid Cymbidium tortisepalum
Source: Int J Mol Sci. 2023 Nov 22;24(23):16627. doi: 10.3390/ijms242316627 (PMC10706266; doi:10.3390/ijms242316627)
Supplement: Supplementary file 1 [file ijms-24-16627-s001.zip › ijms-2618919 - Supplementary.pdf]

Supplementary Information

Sex-Related Gene Network Revealed by Transcriptome Differentiation of Bisexual and Unisexual Flowers of Orchid *Cymbidium tortisepalum*

Xiaokai Ma [1,2,*], Song Ju [1,2], Han Lin [1,2], Huaxing Huang [1], Jie Huang [2], Donghui Peng [2], Ray Ming [1,3], Siren Lan [2] and Zhong-Jian Liu [2,*]

1 Center for Genomics and Biotechnology, Haixia Institute of Science and Technology, School of Future Technology, Fujian Agriculture and Forestry University, Fuzhou 350002, China
2 Key Laboratory of Orchid Conservation and Utilization of National Forestry and Grassland Administration at College of Landscape Architecture, Fujian Agriculture and Forestry University, Fuzhou 350002, China
3 Department of Plant Biology, University of Illinois at Urbana-Champaign, Urbana, IL 61801-3707, USA
* Correspondence: maxk@fafu.edu.cn (X.M.); zjliu@fafu.edu.cn (Z.-J.L.)

Supplementary Figures

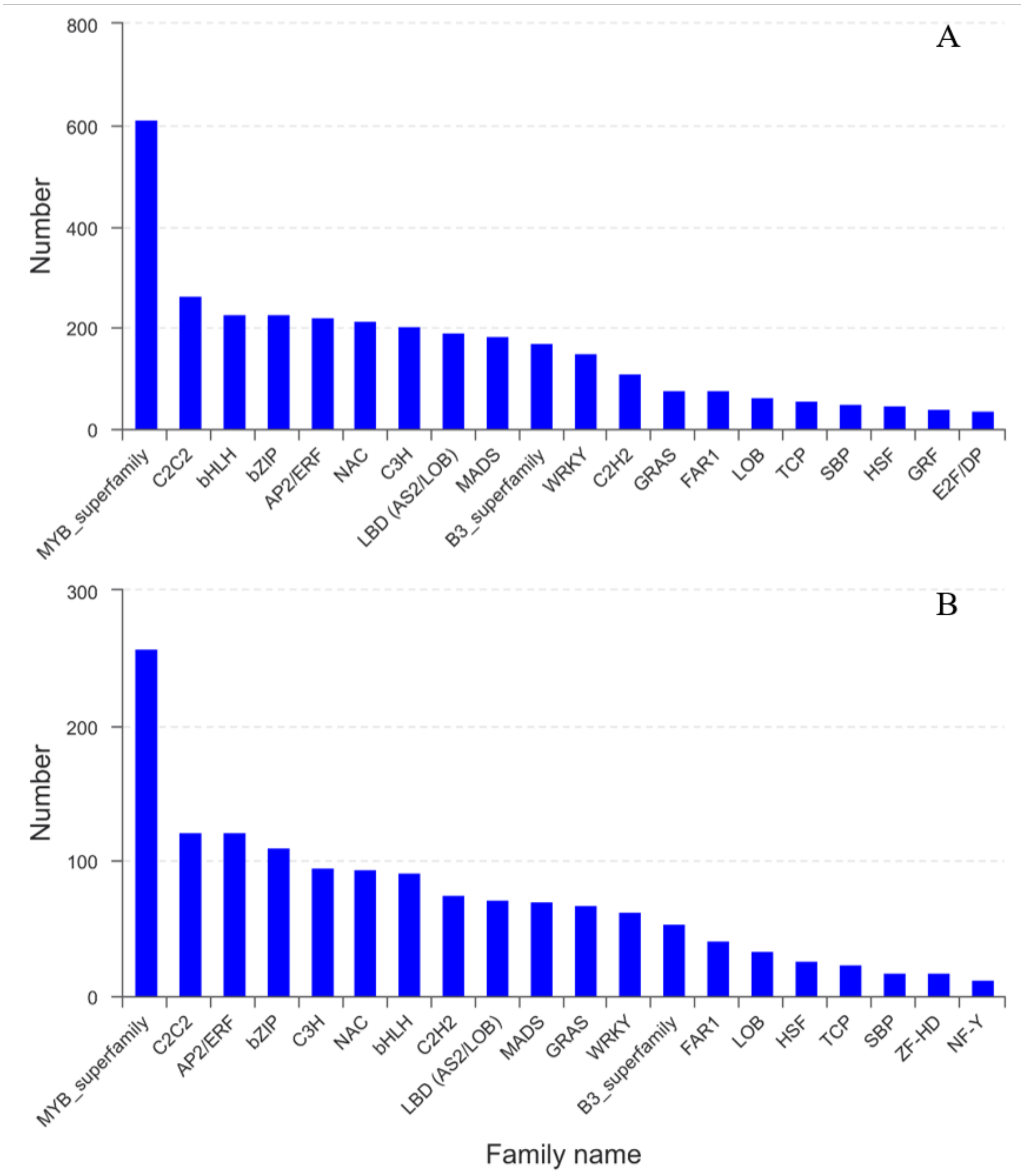

Figure S1. Distribution of identified transcription factors in COOV samples of *C. tortisepalum*. (A) Transcript; (B) Unigenes**.**

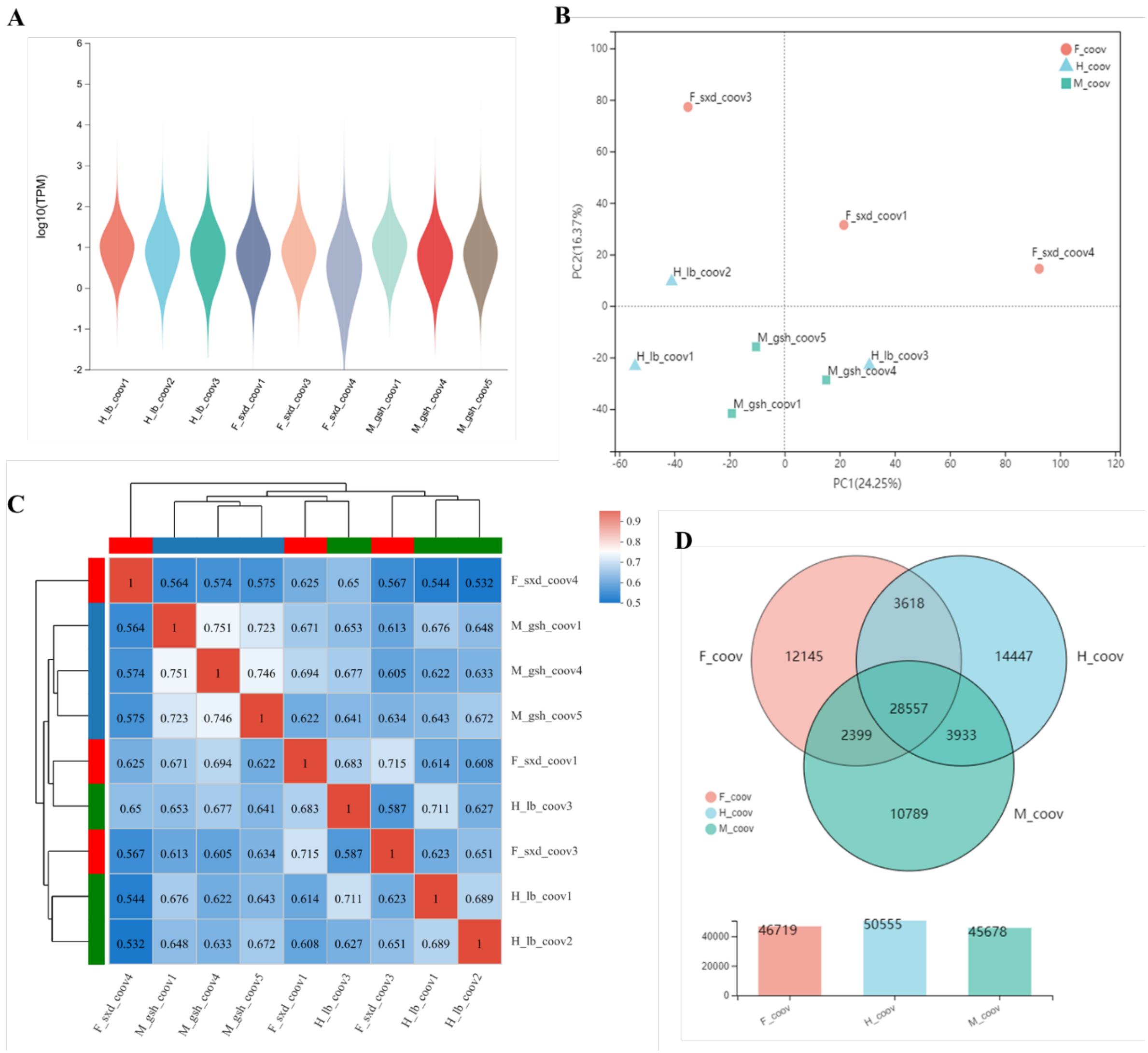

Figure S2. Expression profiles of COOV (mix of gynostemium and ovary) samples among three *C. tortisepalum* sexual phenotypes female (F), male (M) and hermaphrodite bisexual (H) flowers. (A) Distribution of gene expression level in COOV samples among three sexual phenotypes (H-COOV, F-COOV, M-COOV). (B) PCA analysis of unigenes expression profiles for different samples among three sexual phenotypes. (C) Expression correlation matrix among samples of three sexual phenotypes. (D) Venn diagram of gene expression levels in COOV samples for three sexual phenotypes.

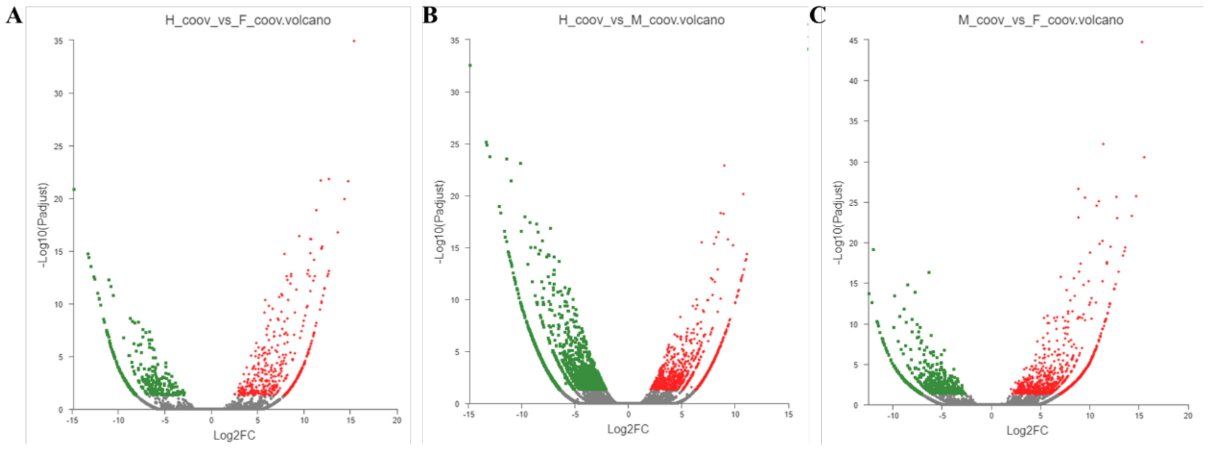

Figure S3. Volcanic map of DEGs in COOV (mix samples of gynostemium and ovary) samples for comparisons of different *C. tortisepalum* sexual phenotypes. (A) Hermaphrodite vs. Female; (B) Hermaphrodite vs. Male; and (C) Male vs. Female.

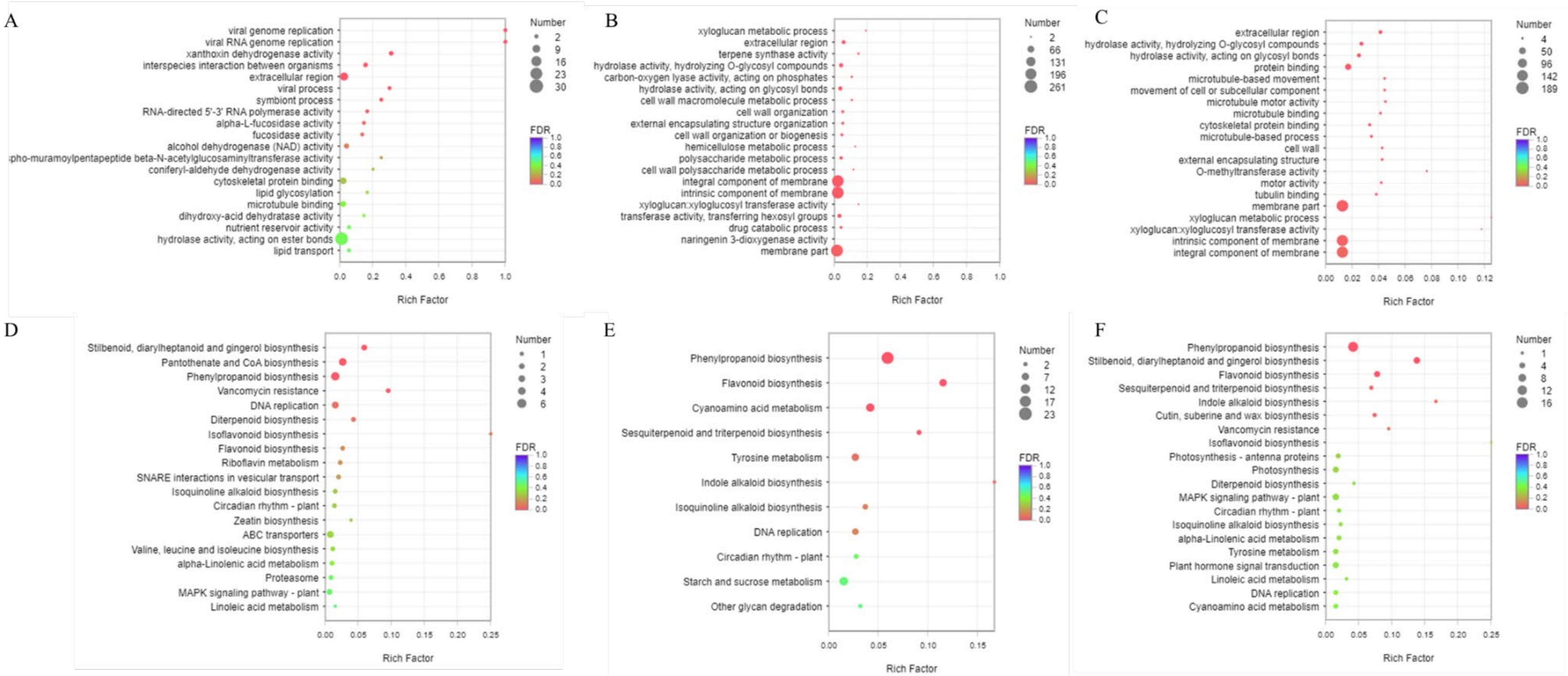

Figure S4. GO and KEGG enrichment of DEGs in the COOV samples for different *C. tortisepalum* sexual phenotypes. GO enrichments for Hermaphrodite vs. Female (A), Hermaphrodite vs. Male (B), and Male vs. Female (C); KEGG enrichments Hermaphrodite vs. Female (D), Hermaphrodite vs. Male (E), and Male vs. Female (F).

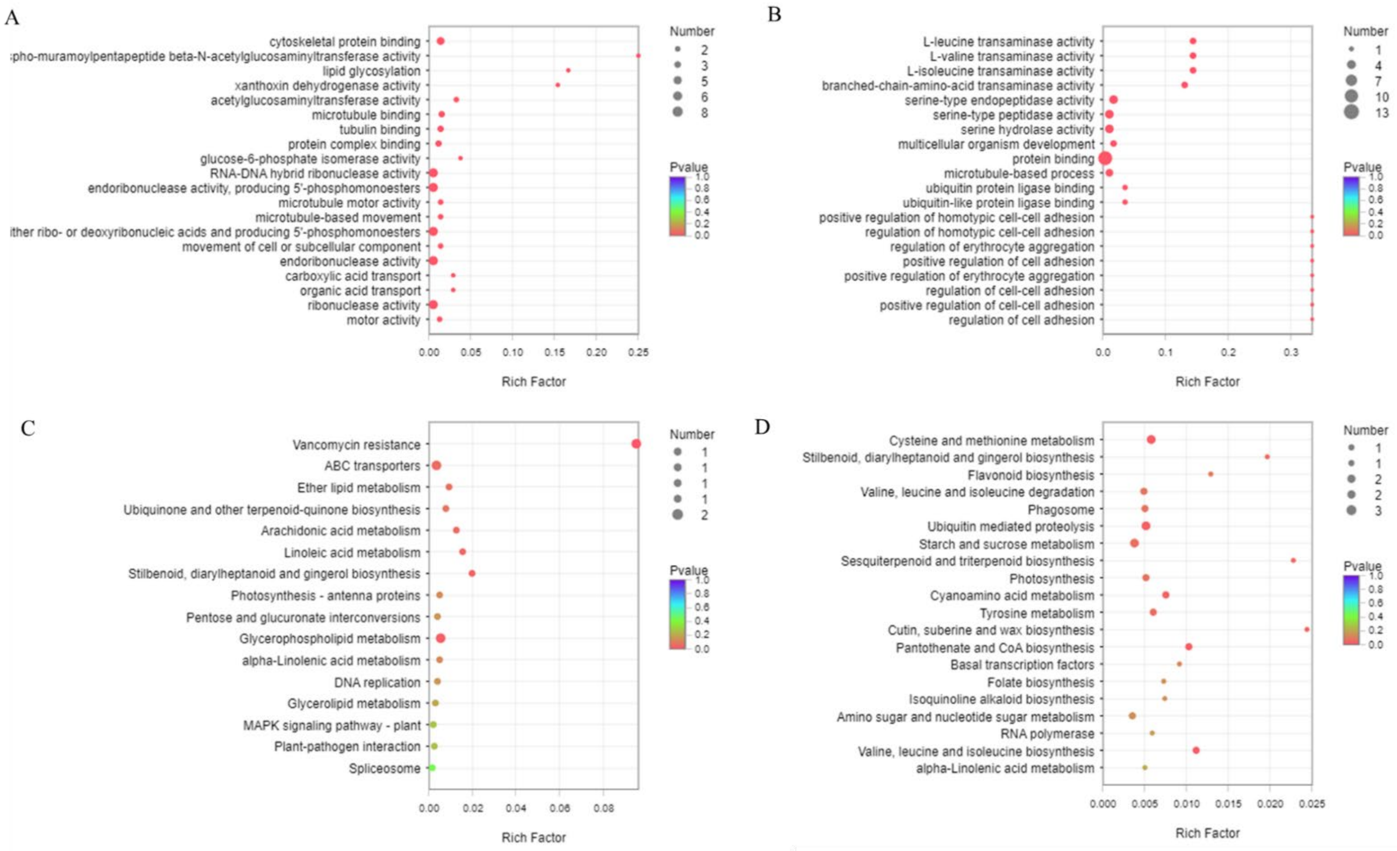

Figure S5. GO and KEGG enrichments of upregulated DEGs in the COOV (mix of gynostemium and ovary) samples for different *C. tortisepalum* sexual phenotypes. GO enrichments for upregulated intersected DEGs between H-COOV vs. F-COOV and M-COOV vs. F-COOV (A); intersected DEGs between H-COOV vs. M-COOV and M-COOV vs. F-COOV (B). KEGG enrichments for upregulated intersected DEGs between H-COOV vs. F-COOV and M-COOV vs. F-COOV (C); intersected DEGs between H-COOV vs. M-COOV and M-COOV vs. F-COOV (D)
